# Fall Risk-Increasing Drugs, Polypharmacy, and Falls Among Low-Income Community-Dwelling Older Adults

**DOI:** 10.1093/geroni/igab001

**Published:** 2021-01-08

**Authors:** Kenya Ie, Eric Chou, Richard D Boyce, Steven M Albert

**Affiliations:** 1 Division of General Internal Medicine, Department of Internal Medicine, St. Marianna University School of Medicine, Kanagawa, Japan; 2 Division of General Internal Medicine, Department of Internal Medicine, Kawasaki Municipal Tama Hospital, Kanagawa, Japan; 3 Department of Biomedical Informatics, University of Pittsburgh, Pennsylvania, USA; 4 Department of Behavioral and Community Health Sciences, University of Pittsburgh Graduate School of Public Health, Pennsylvania, USA

**Keywords:** Falls risk, Medication exposure, Pharmacoepidemiology, Prescription guidance

## Abstract

**Background and Objectives:**

Medication exposure is a potential risk factor for falls and subsequent death and functional decline among older adults. However, controversy remains on the best way to assess medication exposure and which approach best predicts falls. The objective of the current study was to examine the association between different measures of medication exposure and falls risk among community-dwelling older adults.

**Research Design and Methods:**

This retrospective cohort study was conducted using Falls Free PA program data and a linked prescription claims data from Pennsylvania’s Pharmaceutical Assistance Contract for the Elderly program. Participants were community-dwelling older adults living in Pennsylvania, United States. Three measures of medication exposure were assessed: (a) total number of regular medications (polypharmacy); (b) counts of potentially inappropriate medications derived from current prescription guidance tools (Fall Risk-Increasing Drugs [FRIDs], Beers Criteria); and (c) medication burden indices based on pharmacologic mechanisms (Anticholinergic Cognitive Burden, Drug Burden Index) all derived from claims data. The associations between the different medication risk measures and self-reported falls incidence were examined with univariate and multivariable negative binomial regression models to estimate incidence rate ratios (IRRs).

**Results:**

Overall 343 older adults were included and there were 236 months with falls during 2,316 activity-adjusted person-months (10.2 falls per 100 activity-adjusted person-months). Of the 6 measures of medication risk assessed in multivariate models, only the use of 2 or more FRIDs (adjusted IRR 1.67 [95% CI: 1.04–2.68]) independently predicted falls risk. Among the 13 FRID drug classes, the only FRID class associated with an increased fall risk was antidepressants.

**Discussion and Implications:**

The presence of multiple FRIDs in a prescription is an independent risk factor for falls, even in older adults with few medications. Further investigation is required to examine whether deprescribing focused on FRIDs effectively prevents falls among this population.

**Translational Significance:** The current study examined different medication exposure measures to see which best predicts falls among community-dwelling older adults. The study found that the presence of multiple Fall Risk-Increasing Drugs (FRIDs) in a prescription is an independent risk factor for falls, even in people with few medications. This result has important clinical implications for future deprescribing strategies, where focusing on FRIDs would be more effective than simply reducing the number of medications.

## Background and Objectives

The frequency of falls increases as people age. According to the Behavioral Risk Factor Surveillance System survey, approximately 29% of people aged 65 and older experienced an accidental fall in any given year (1). Accidental falls and fall-related injuries among this population have been shown to result in significant morbidity and mortality (2–4). Thus, along with the increasing aging population, falls prevention has been a major public health priority.

Falls occur as a result of a complex interaction of numerous risk factors: age, sex, comorbidities, previous falls, functional dependency, and medication burden are previously described potential risk factors (5,6). Among potential risk factors for falls, several risk factors such as medication exposure, excess alcohol use, and sedentary lifestyle are potentially modifiable. It is important to further elucidate modifiable fall risk factors among older adults so as to inform evidence-based public health interventions.

Risks from medication exposure can be defined in a number of ways. One is simply a count of medications, or polypharmacy. Although no standard definition has been set, polypharmacy is often defined as regular use of five or more medications. Several previous studies showed that polypharmacy was associated with falls risk (7–9) and impaired balance (10) among older populations. On the contrary, other authors reported that polypharmacy itself was not an independent risk factor for accidental falls after controlling for other variables such as age, sex, and comorbidities (11,12). A recent systematic review regarding health outcomes associated with polypharmacy reported that 19 out of 23 studies found at least one positive association between polypharmacy and either falls or fall-related outcomes. However, the authors questioned whether the number of medications prescribed itself is an independent risk factor for falls or whether the association could be explained by the fact that the exposure to Fall Risk-Increasing Drugs (FRIDs) is likely to be present as a result of polypharmacy (13). In addition, polypharmacy can be appropriate, especially in the management of older patients with multimorbidity.

A second approach defines medication exposure by a count of potentially inappropriate medications (PIMs), which have been specified in prescribing guidance tools. These tools include the Beers (14) and the STOPP/START criteria (15). More recently, the Swedish National Board of Health and Welfare developed prescribing guidance for FRIDs (16). The list includes five drug classes under “drugs that cause high risk of falling” (e.g., opioids and antipsychotics) and eight drug classes under “drugs that cause orthostatism/hypotension” (e.g., diuretics and beta-blockers). Each of these prescribing guidelines identifies PIMs, and a count of these medications is sometimes used as a measure of medication exposure risk (6,17).

Finally, explicit medication burden measures have been proposed to assess the cumulative effect of anticholinergic and sedative properties. These have proven useful in the field of pharmacoepidemiology. The Drug Burden Index (DBI) measures overall exposure to medications with anticholinergic (DBI-Ach) and sedative properties (DBI-Se) (18). Another widely used tool, the Anticholinergic Cognitive Burden (ACB) (19), focuses on cognitive effects of medications with anticholinergic activity.

To date, controversy remains regarding which approach is best for determining falls risk among older adults. In this research we examined (a) polypharmacy, (b) counts of PIMs specified by the Beers Criteria and Swedish National Board of Health and Welfare FRIDs prescription guidance tool, and (c) medication burden measures as alternative predictors of falls risk in a community sample.

We hypothesized that explicit medication burden measures such as ACB and DBI, which incorporate cumulative burden of medication exposure, would have better predictive capacity compared to the simple count of regular medications or prescription guidance based on PIMs. The objectives of the current study were to examine the association between different medication exposure measures and falls and to determine which best predicts falls among community-dwelling older adults.

## Research Design and Methods

### Study Design and Setting

This retrospective cohort study examined the risk of falls according to medication exposure among community-dwelling older adults in Pennsylvania, United States. Falls Free PA program data were linked to prescription claims data from Pennsylvania’s Pharmaceutical Assistance Contract for the Elderly (PACE) program. Falls Free PA is a previously conducted cohort study comparing falls incidence between participants of Pennsylvania’s Healthy Steps for Older Adults program and a control group (20).

### Study Sample

**Figure 1.**
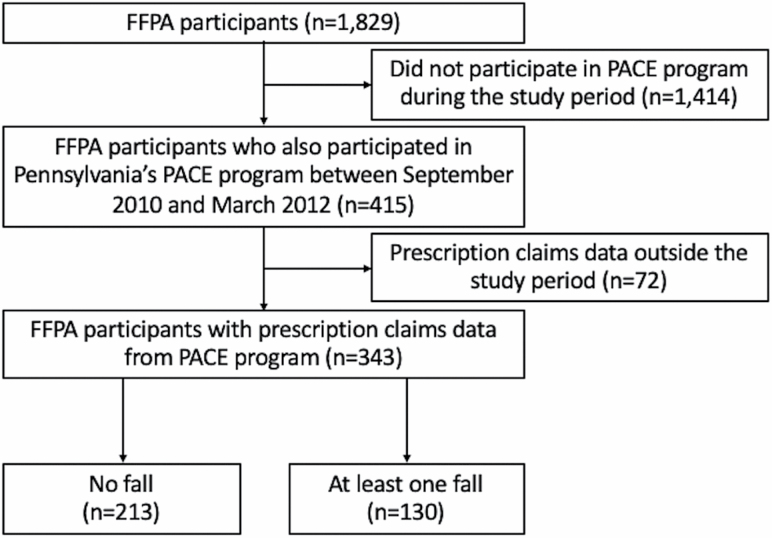
Flow diagram of participant selection. *Notes*: FFPA = Falls Free PA; PACE = Pennsylvania’s Pharmaceutical Assistance Contract for the Elderly.

The original Falls Free PA included a total of 1,829 older adults who had participated in senior center activities across 19 Pennsylvania counties from 2010 to 2011. Among the Falls Free PA participants, our analysis included those who also participated in Pennsylvania’s PACE program between September 2010 and March 2012. PACE eligibility criteria (21) include (a) adults aged 65 years or older; (b) Pennsylvania residency for at least 90 days; (c) not being enrolled in the Medicaid prescription benefit; and (d) a total annual income of $14,500 or less (for a married couple combined total annual income must be $17,700 or less). Exclusion criteria were language use other than English or Spanish, and inability to participate in telephone follow-up calls (20). The patient selection flow diagram is shown in Figure 1.

### Outcome Measurements

The primary outcome was self-reported falls during one of the monthly telephone interviews that occurred during the 12-month Falls Free PA cohort study. The incidence rate of fall-months was calculated as self-reported months in which participants reported at least one fall, per 100 person-months of follow-up, adjusted for participants’ activity levels. Interviews with Falls Free PA program participants who had fallen revealed that 89% of fall-months involved a single fall. Accordingly, we used fall-months per 100 person-months to indicate incidence. We adjusted follow-up time using the number of days participants reported physical activity during follow-up. This decision was based on evidence that older people with mobility limitations may reduce their activities to minimize the fall risk (22). We defined “physically active” as participating in moderate or vigorous activities for at least 30 min on a day (20).

### Medication Exposure Variables

We used study participants’ PACE prescription claims data between the baseline and the 12-month follow-up to measure risks from medication exposure. The medication data gathered from prescription claims included drug name, daily dose, form, and days supply. All forms of oral, suppository, and transdermal medications were included in the current analysis. The FRIDs list proposed by the Swedish National Board of Health and Welfare (16) was used as the primary exposure of interest, utilizing the Anatomical Therapeutic Chemical classification system (Supplementary Table 1). Medications were considered to be “regular” for an individual participant when claims for a specific medication for the participant were provided 30 days or longer at least twice during the study period. For the current study, the number of regular medications was categorized into 0, 1–4, and 5 or more medications, with people with no medications serving as the reference group. We defined “polypharmacy” as the prescription of five or more “regular medications.” Likewise, categories were established based on a number of regular FRIDs of 0, 1, and 2 or more. Patients who took no FRIDs served as the reference group. The number of medications recommended to be avoided for older adults in table 2 of the Beers Criteria (23) was also counted and dichotomized into 0 and 1 or more for medications in the Beers Criteria. Drugs that require prescription indications (e.g., opioids: avoid, excludes pain management due to recent fractures or joint replacement) in the Beers Criteria were not measured because of data limitations. Other exposure variables to quantify the medication burden included the ACB (19) and the DBI (18). The drug class overlap between FRIDs, ACB, DBI, and the Beers Criteria are summarized in Supplementary Table 2. The cumulative medication burden of ACB, Drug Burden Index—sedative property (DBI-Se), and DBI-Ach was quantified based on the following equations (24,25) and split into categories of 0, low, and high burden, with cutoff values between low and high burden being the median values among those with scores above 0:

$$\begin{array}{l} ACB_{i}=\frac{\sum_{ia=i1}^{ik}\left(ACB\;weight\;for\;drug\;a*Days\;Supply\right)}{Days\;\;between\;Baseline\;and\;12\;month} \end{array}$$

*i*: subject; *a*: drug name listed in ACB list.

$$\begin{array}{l} DBI\;\_\;Se_{i}=\frac{\sum_{is=i0}^{ik}\left(\frac{D_{s}}{\delta_{s}+D_{s}}\;\;*Days\;Supply\;\;s\right)}{Days\;\;between\;\;Baseline\;\;and\;12\;month} \end{array}$$

*s*: drug name listed in DBI-Se list; *D*: daily dose of the drug; δ: minimum effective dose of the drug.

$$\begin{array}{l} DBI\;\_\;Ach_{i}=\frac{\sum_{ib=i1}^{ik}\left(\frac{D_{b}}{\delta_{b}\;+\;D_{b}}\;\;*Days\;Supply\;b\right)}{Days\;\;between\;\;Baseline\;\;and\;12\;month} \end{array}$$

*b*: drug name listed in DBI-ACh list.

### Covariates

At baseline, other variables measured included age, gender, race, living status, education level, income level, falls in the preceding year, self-rated mobility, medical conditions, the EuroQoL five-dimension three-level (EQ-5D-3L) summary index (26), and memory performance (Memory Impairment Screen—Telephone [MIS] (27)).

### Statistical Analyses

A univariate negative binomial regression model was used to estimate incidence rate and 95% confidence interval (CI) for fall-months stratified by the medication exposure measures and to assess potential associations between each measure and falls risk. The associations between each medication exposure and falls incidence were examined with separate multivariable negative binomial regression models, adjusting for age, gender, baseline MIS score, baseline self-rated mobility, and falls in the preceding year. Activity-adjusted follow-up was used as an offset variable to give incidence rate ratios (IRRs). Multicollinearity was tested based on the variance inflation factor, with a cutoff at 10. Subgroup analyses of FRIDs on the risk of falls by FRID classes were evaluated adjusting for age, gender, and total number of regular medications. We used a significance level of .05 for hypothesis testing. All statistical analyses were performed using STATA/SE 15.0 (StataCorp LLC, College Station, TX).

## Results

Among 415 older adults included in the Falls Free PA and Pennsylvania’s PACE program with fall incidence data, 72 were excluded from the current analysis due to missing data regarding prescription claims data, leaving 343 older adults. Their mean (*SD*) age was 78.3 (6.6) years, 35 (10.2%) were men, 257 (74.8%) lived alone, 104 (30.3%) used an assistive device for walking, and 108 (31.6%) reported that they had fallen at least once within the year prior to the research. The mean (*SD*) number of comorbidities among the 17 indicator chronic diseases was 3.8 (1.8), and the most common comorbidity was hypertension (*n* = 259, 75.5%) followed by arthritis (*n* = 248, 72.3%). The number with one or more medications identified with each specific medication exposure measure was 254 (74.1%) for FRIDs, 102 (29.7%) for ACB, 168 (49.0%) for DBI-total, 126 (36.7%) for DBI-Se, 82 (23.9%) for DBI-ACh, and 66 (19.2%) for the Beers Criteria. The mean (*SD*) number of prescribed regular medications were 3.99 (3.07) among those who did not fall during the follow-up and 4.33 (2.87) among fallers. Compared to nonfallers, participants with one or more falls were more likely to have fallen in the year before study participation and had lower baseline memory score and EQ-5D score (Table 1).

**Table 1.** Baseline Characteristics of Study Participants (*N* = 343)

Characteristics	Participants without fall-month (*n* = 213)	Participants with ≧1 fall-months (*n* = 130)	*p* Value^a^
Age—mean (*SD*)	77.9 (6.7)	79.0 (6.4)	.13
Gender (male, %)	19 (8.9%)	16 (12.3%)	.32
Race (%)			.02
White	201 (94.8%)	113 (86.9%)	
Black	11 (5.2%)	15 (11.5%)	
Others	0 (0%)	2 (1.6%)	
Live with someone (yes, %)	51 (24.1%)	35 (27.1%)	.53
Education (college or more, %)	44 (20.7%)	40 (30.8%)	.04
Income (sufficient for daily living, %)	164 (80.0%)	101 (80.8%)	.86
Fall in the previous year (yes, %)	49 (23.1%)	59 (45.4%)	<.01
Use of assistive devices (yes, %)	59 (27.7%)	45 (34.6%)	.18
Number of comorbidity (*SD*)	3.7 (1.9)	4.0 (1.7)	.10
Memory score (*SD*) (range 0–8)	6.4 (1.4)	6.1 (1.6)	.04
Self-rated mobility (*SD*) (range 1–5)^b^	2.6 (0.9)	2.9 (1.0)	.01
EQ-5D index (*SD*) (range 0–1)	0.84 (0.01)	0.80 (0.01)	.01
Medication-related variables			
Number of regular medications (*SD*)^c^	3.99 (3.07)	4.33 (2.87)	.31
Number of regular FRIDs (*SD*)^c^	1.54 (1.42)	1.65 (1.28)	.47
Number of Beers Criteria medications (*SD*)^c^	0.23 (0.55)	0.24 (0.49)	.95
Cumulative ACB score (*SD*)	0.55 (0.91)	0.57 (0.81)	.84
Cumulative DBI-Se score (*SD*)	0.10 (0.22)	0.15 (0.27)	.04
Cumulative DBI-Ach score (*SD*)	0.05 (0.15)	0.06 (0.13)	.61

*Notes*: ACB = Anticholinergic Cognitive Burden; DBI-Ach = Drug Burden Index—anticholinergic property; DBI-Se = Drug Burden Index—sedative property; EQ-5D = EuroQOL 5 dimensions; FRIDs = Fall Risk-Increasing Drugs.

^a^ *p* Values are for the comparison between fallers and nonfallers.

^b^1 = excellent; 5 = poor.

^c^Number of drugs prescribed at least 30 days for at least twice during the study period.

Of the 343 participants, 152 (44.3%) reported at least one fall-month during the study period. There were 236 months with falls during 2,316 activity-adjusted person-months, which is equivalent to 10.2 fall-months per 100 person-months (95% CI: 10.07–10.33). The fall risk per 100 activity-adjusted person-months based on the different medication risk measure strata are shown in Table 2.

**Table 2.** Medication Exposure and the Fall Risk Per 100 Activity-Adjusted Person-Months

Medication exposure	*N*	Number of falls (months)	Person-months adjusted for activity	Fall-months incidence rate per 100 person-months of activity-adjusted follow-up
Number of regular medications				
0	40	15	268.09	5.60 (3.13–9.23)
1–4	166	115	1,153.93	9.97 (8.23–11.96)
5+	137	106	893.64	11.86 (9.71–14.35)
Number of FRIDs				
0	89	42	592.59	7.09 (5.11–9.58)
1	90	64	640.21	10.00 (7.70–12.77)
2+	164	130	1,082.86	12.01 (10.03–14.26)
Number of Beers Criteria medications				
0	277	191	1,862.56	10.25 (8.85–10.40)
≥1	66	45	453.1	9.93 (7.24–13.29)
Cumulative ACB score^a^				
0	141	88	967.12	9.10 (7.30–11.21)
Low ACB score	100	60	665.58	9.01 (6.88–11.60)
High ACB score	102	88	682.96	12.86 (10.33–15.87)
Cumulative DBI—sedative property score^a^				
0	217	136	1,500.74	9.06 (7.60–10.72)
Low DBI-Se score	61	44	364.81	12.06 (8.76–16.19)
High DBI-Se score	65	56	450.11	12.44 (9.40–16.16)
Cumulative DBI—anticholinergic property score^a^				
0	261	176	1,798.5	9.79 (8.39–11.34)
Low DBI-Ach score	41	19	256.78	7.40 (4.45–11.55)
High DBI-Ach score	41	41	260.38	15.75 (11.30–21.36)
**Total**	343	236	2,315.66	10.20 (8.93–11.58)

*Notes*: ACB = Anticholinergic Cognitive Burden; DBI-Ach = Drug Burden Index—anticholinergic property; DBI-Se = Drug Burden Index—sedative property; FRIDs = Fall Risk-Increasing Drugs; IRR = incidence rate ratio.

^a^The cutoff values between the low and high scores were determined to be median values among those higher than 0 (ACB: 0.655, DBI-Se: 0.233, DBI-Ach: 0.150).

Of the six medication risk measures, only FRIDs showed a statistically significant dose effect in models adjusting for age, gender, and baseline mobility score. The adjusted IRR (95% CI) for 2+ FRIDs was 1.67 (1.04–2.68; Table 3). Polypharmacy (five or more regular medications) was marginally significant as a falls risk factor after adjusting for these baseline potential confounders (adjusted IRR 1.92 [95% CI: 0.94–3.92]). Other cumulative medication burden scales of ACB (19) and DBI (18), as well as the Beers Criteria (23), did not predict falls after adjusting for covariates. To assess the relative role of polypharmacy and FRIDs for falls risk, we conducted additional stratified analyses according to the presence or absence of two or more FRIDs in the medication list. Taking two or more FRIDs at baseline revealed a nonsignificant increase in the risk of falls among older adults with 0–4 regular medications, whereas FRIDs were no longer a falls risk among those with five or more regular medications (Table 4).

**Table 3.** The Association of Medication Exposure and Falls Incidence Among Community-Dwelling Older Adults

Medication exposure	Unadjusted incidence rate ratio for fall-months	Adjusted incidence rate ratio for fall-months^a^
Number of regular medications		
0	Ref	Ref
1–4	1.69 (0.84–3.42)	1.65 (0.81–3.34)
5+	2.08 (1.02–4.25)*	1.92 (0.94–3.92)
Number of FRIDs		
0	Ref	Ref
1	1.37 (0.80–2.36)	1.44 (0.84–2.45)
2+	1.69 (1.04–2.74)*	1.67 (1.04–2.68)*
Number of Beers Criteria medications		
0	Ref	Ref
≥1	1.01 (0.62–1.64)	1.15 (0.72–1.84)
Cumulative ACB score		
0	Ref	Ref
0 < ACB ≦ 0.655^b^	0.96 (0.60–1.54)	1.00 (0.63–1.57)
0.655 <	1.41 (0.90–2.20)	1.24 (0.80–1.92)
Cumulative DBI—sedative property score		
0	Ref	Ref
0 < DBI-Se ≦ 0.233^b^	1.45 (0.87–2.42)	1.32 (0.80–2.18)
0.233 <	1.40 (0.87–2.26)	1.30 (0.82–2.06)
Cumulative DBI—anticholinergic property score		
0	Ref	Ref
0 < DBI-Ach ≦ 0.15^b^	0.70 (0.37–1.33)	0.52 (0.27–1.01)
0.15 <	1.76 (1.02–3.04)^‡^	1.51 (0.88–2.58)

*Notes*: ACB = Anticholinergic Cognitive Burden; DBI = Drug Burden Index; FRIDs = Fall Risk-Increasing Drugs.

^a^Adjusted for age, gender, falls in the preceding year, baseline memory score, and baseline mobility score.

^b^The cutoff values were determined to be median values among those higher than 0.

*p < .05.

**Table 4.** Adjusted Incidence Rate Ratio of Falls Stratified by Number of Regular Medications and FRID

Medication exposure	*n*	Number of fall-months	Person-months adjusted for activity	Fall-months incidence rate per 100 person-months of activity-adjusted follow-up	Adjusted incidence rate ratio for fall-months^a^
Number of regular medications 0–4 (*n* = 206)					
0–1 FRID	156	90	1,090.87	8.25 (6.63–10.14)	Ref
2+ FRIDs	50	40	331.15	12.08 (8.63–16.45)	1.43 (0.84–2.43)
Number of regular medications 5+ (*n* = 137)					
0–1 FRID	23	16	141.93	11.27 (6.44–18.31)	Ref
2+ FRIDs	114	90	751.71	11.97 (9.63–14.72)	0.96 (0.44–2.11)

*Notes*: FRIDs = Fall Risk-Increasing Drugs.

^a^Adjusted for age, gender, falls in the preceding year, baseline memory score, and baseline mobility score.

The five most common regular FRID classes were agents acting on the renin–angiotensin system (42.9%), beta-blockers (28.9%), diuretics (23.9%), calcium channel blockers (23.0%), and antidepressants (16.3%). Among the 13 FRID classes, the only FRID class associated with an increased fall risk was antidepressants. This association remained significant after adjusting for age, gender, and total number of regular medications (adjusted IRR 1.71 [95% CI: 1.05–2.78]). Other FRID classes including cardiovascular medications and central nervous system medications revealed no significant association with falls (Table 5).

**Table 5.** Incidence Rate Ratio (IRR) of Falls for the 13 FRID Classes Among Older Adults (Exploratory Analysis)

FRIDs drug class	Fallers (*N* = 130)	Nonfallers (*N* = 213)	Crude IRR	Adjusted IRR^a^
Drugs that cause high risk of falling				
Opioids (%)	1 (0.77)	6 (2.8)	0.24 (0.03–2.25)	0.19 (0.02–1.80)
Antipsychotics (%)	0 (0)	3 (1.4)	—	—
Anxiolytics (%)	11 (8.5)	21 (9.9)	0.75 (0.38–1.51)	0.69 (0.34–1.37)
Hypnotics and sedatives (%)	4 (3.1)	7 (3.3)	0.56 (0.16–2.01)	0.49 (0.14–1.75)
Antidepressants (%)	32 (24.6)	24 (11.3)	1.85 (1.15–2.96)*	1.71 (1.05–2.78)*
Drugs that cause orthostatism/hypotension				
Vasodilators used in cardiac diseases (%)	3 (2.3)	6 (2.8)	1.40 (0.45–4.32)	1.22 (0.40–3.70)
Diuretics (%)	30 (23.1)	52 (24.4)	1.27 (0.82–1.97)	1.03 (0.63–1.68)
Beta-blockers (%)	40 (30.8)	59 (27.7)	1.32 (0.87–1.98)	1.10 (0.71–1.72)
Calcium channel blockers (%)	31 (23.8)	48 (22.5)	1.38 (0.89–2.14)	1.16 (0.73–1.84)
Agents acting on the renin–angiotensin system (%)	52 (40.0)	95 (44.6)	0.97 (0.66–1.42)	0.81 (0.53–1.22)
Alpha-adrenoreceptor antagonists (%)	3 (2.3)	2 (0.9)	1.39 (0.30–6.37)	0.99 (0.20–4.85)
Other antihypertensives (%)	3 (2.3)	3 (1.4)	0.96 (0.23–4.08)	0.77 (0.18–3.37)
Anti-Parkinson drugs (%)	5 (3.8)	3 (1.4)	1.91 (0.66–5.55)	1.56 (0.53–4.58)

*Notes*: FRIDs = Fall Risk-Increasing Drugs.

^a^Adjusted for age, gender, and number of regular medications.

*p < .05.

## Discussion and Implications

In this retrospective cohort study, the incidence of falls among low-income community-dwelling older adults increased with greater medication exposure across all three types of exposure. However, in multivariable models that adjusted for other falls risk factors, only the use of two or more FRIDs remained a significant predictor. Polypharmacy (five or more regular medications) was only marginally associated with falls risk after adjusting for age, gender, baseline MIS score, baseline self-rated mobility, and falls in the preceding year. Similar to our results, Zia et al. have reported that the use of two or more FRIDs independently predicted recurrent and injurious falls (6). Likewise, Bennett et al. revealed an association between the number of FRIDs on discharge and a greater risk of recurrent falls (17). It has been argued that the harm of polypharmacy may not consistently outweigh its benefit, depending on the appropriateness of medications prescribed (28). At the same time, polypharmacy is known to be associated with inappropriate prescribing (13). Thus, it is possible that polypharmacy increases the risk of FRIDs exposure that poses an increased risk of falling. Our results imply that the effect of polypharmacy on falls risk is likely mediated by the presence of FRIDs. The inconsistent association of polypharmacy with falls in previous studies may result from not taking into account the number of FRIDs in counts of medications.

As stated before, we hypothesized that state-of-the-art medication burden indices, such as the cumulative ACB and cumulative DBI, would be superior to polypharmacy and PIMS based on prescribing guidance, such as the FRIDs list and Beers Criteria. Although evidence is limited regarding such head-to-head comparisons, the association between medication burden scales, such as higher DBI scores and decreases in physical function among older adults, suggests such an association (29,30). However, previous research has yielded conflicting evidence regarding the effect of DBI on falls risk. A study conducted by Wilson et al. revealed a significant association between DBI and falls in older adults living in residential aged care facilities (31). More recently, Cardwell et al. reported that a higher DBI score was associated with a greater risk of mortality but not with an increased rate of falls among older participants of a cohort study in New Zealand (32). Our results imply that the simple count of FRIDs, as well as the number of regular medications, could be at least as effective as the more complex medication burden measurements in measuring falls risk among older adults. More evidence is required to confirm the utility of FRIDs for screening and intervention of older adults at risk for falls.

Based on our stratified analyses, polypharmacy was not a significant falls risk in models that stratified for the presence of two or more FRIDs. Thus, we infer that exposure to FRIDs is important for the polypharmacy–falls relationship. This inference is supported by a large-scale cross-sectional study conducted in the Netherlands that revealed an association between polypharmacy and falls only when falls risk-increasing medications were included in prescriptions (33). Further investigations with larger sample sizes are required to confirm whether the number of medications prescribed itself has some effect on falls risk when FRIDs are prescribed.

When examining individual FRID classes and falls incidence, antidepressants was the only FRID class with an increased falls risk. Our finding is consistent with findings by Kuschel et al., who reported that central nervous system drugs including antidepressants showed an increased risk of falls (34). In our analysis, other central nervous system drugs were not significant for falls risk, potentially due to the relatively small number of prescriptions involving these drugs. This may result from the fact that the PACE program generally does not encourage clinicians to prescribe high-risk medications. In addition, our analysis examined all falls rather than injurious falls or recurrent falls, which is often the outcome for research on falls. It is noteworthy that another study of community-dwelling older adults found antidepressants to be associated with recurrent falls in community-dwelling older adults (adjusted odds ratio: 1.48; 95% CI = 1.12–1.96) (35).

Several limitations of our study need to be considered. Our data did not include over-the-counter medications, which could also contain sedative and anticholinergic properties. Assuming that multimorbid older adults with polypharmacy are more likely to take over-the-counter medications, our results may overestimate the effect of prescribed FRIDs. In addition, our data set did not include information regarding medication adherence, which is a crucial aspect of pharmacoepidemiology studies. Secondly, we found increased effect sizes for all of the medication measures, except for the Beer’s Criteria, which were not significant possibly due to the small sample. Thus, we are unable to rule out that some of these other measures are also related to falls. Finally, PACE program features (e.g., eligibility based on low income) require caution in generalizing our findings to the population of community-dwelling older adults.

The major finding of our results is that the presence of multiple FRIDs in a prescription is an independent risk factor for falls, even in people with few medications. Despite the growing body of evidence regarding the association between medication exposure and falls, there is limited evidence available for the effect of reducing FRIDs. A few studies have demonstrated the potential benefit of deprescribing on falls prevention (36,37), while other randomized controlled trials have failed to demonstrate clinical benefit (38,39). Thus, future studies with sufficient statistical power will be required to confirm whether deprescribing focused on FRIDs, either overall or for specific FRID classes, effectively prevents falls and subsequent adverse health outcomes among older adults.

## Supplementary Material

igab001_suppl_Supplementary_Materials
