# Supplementary material for: Fall Risk-Increasing Drugs, Polypharmacy, and Falls Among Low-Income Community-Dwelling Older Adults
Source: Innov Aging. 2021 Jan 8;5(1):igab001. doi: 10.1093/geroni/igab001 (PMC7899132; doi:10.1093/geroni/igab001)
Supplement: igab001_suppl_Supplementary_Materials [file igab001_suppl_supplementary_materials.docx]

Online Supplemmentary Material for Publication in *Innovation in Aging*:

Fall Risk-Increasing Drugs, Polypharmacy, and Falls among Low-Income Community-Dwelling Older Adults

Kenya Ie, MD, PhD, MPH^1,2^, Eric Chou, BSc^3^, Richard D. Boyce, PhD^3^, and Steven M. Albert, PhD^4^

1. Division of General Internal Medicine, Department of Internal Medicine, St. Marianna University School of Medicine, Kanagawa, Japan.

2. Division of General Internal Medicine, Department of Internal Medicine, Kawasaki Municipal Tama Hospital, Kanagawa, Japan.

3. Department of Biomedical Informatics, University of Pittsburgh, Pittsburgh, Pennsylvania, USA.

4. Department of Behavioral and Community Health Sciences, University of Pittsburgh Graduate School of Public Health, Pittsburgh, Pennsylvania, USA.

*Address correspondence to: Kenya Ie, MD, PhD, MPH, 1-30-37 Shukugawara, Kawasaki, Kanagawa 214-8525, Japan. E-mail: [kenya.ie@marianna-u.ac.jp](mailto:kenya.ie@marianna-u.ac.jp)

Supplementary Table 1. Fall Risk-Increasing Drugs definition^a^

| **FRID Class** | **ATC Codes** |
| --- | --- |
| Drugs that cause high risk of falling | |
| Opioids | N02A |
| Antipsychotics | N05A excluding Lithium |
| Anxiolytics | N05B |
| Hypnotics and sedatives | N05C |
| Antidepressants | N06A |
| Drugs that cause orthostatism/hypotension | |
| Vasodilators used in cardiac diseases | C01D |
| Antihypertensives | C02 |
| Diuretics | C03 |
| Beta-blockers | C07 |
| Calcium channel blockers | C08 |
| Agents acting on the renin-angiotensin system | C09 |
| Alpha-adrenoreceptor antagonists | G04CA |
| Anti-Parkinson drugs | N04B |

Notes. FRIDs=Fall Risk-Increasing Drugs; ATC=Anatomical Therapeutic Chemical classification system.

^a^ Data adapted from: Correa-Pérez A, Delgado-Silveira E, Martín-Aragón S, Cruz-Jentoft AJ. Fall-risk increasing drugs and recurrent injurious falls association in older patients after hip fracture: a cohort study protocol. *Ther Adv drug Saf*. 2019;10(6):2042098619868640. doi:10.1177/2042098619868640

Supplementary Table 2. Overlap between Fall Risk-Increasing Drug classes and other medication risk measures

| **FRIDS Class** | **ATC Code** | **ACB** | **DBI-Se** | **DBI-Ach** | **Beers Criteria** |
| --- | --- | --- | --- | --- | --- |
| Opioids | N02A | * | * |  |  |
| Antipsychotics | N05A excluding Lithium | * | * | * | * |
| Anxiolytics | N05B | * | * | * | * |
| Hypnotics and sedatives | N05C | * | * |  | * |
| Antidepressants | N06A | * | * | * | * |
| Vasodilators used in cardiac diseases (nitrates) | C01D | * |  |  |  |
| Antihypertensives | C02 | * | * |  | * |
| Diuretics | C03 | * |  |  |  |
| Beta-blockers | C07 | * |  |  |  |
| Calcium channel blockers | C08 | * |  |  |  |
| Agents acting on the renin-angiotensin system | C09 | * |  |  |  |
| Alpha-adrenoreceptor antagonists | G04CA | * |  |  | * |
| Dopaminergic agents (anti-Parkinson drugs) | N04B | * |  | * | * |

*Notes*. FRIDs=Fall Risk-Increasing Drugs; ATC=Anatomical Therapeutic Chemical classification system; ACB=Anticholinergic Cognitive Burden; DBI=Drug Burden Index.

*denotes overlap in drug class between FRIDs and other medication risk measures.
